# Interaction Study of Phospholipid Membranes with an *N*-Glucosylated *β*-Turn Peptide Structure Detecting Autoantibodies Biomarkers of Multiple Sclerosis

**DOI:** 10.3390/membranes5040576

**Published:** 2015-09-30

**Authors:** Lucia Becucci, Stefano Benci, Francesca Nuti, Feliciana Real-Fernandez, Zahra Vaezi, Lorenzo Stella, Mariano Venanzi, Paolo Rovero, Anna Maria Papini

**Affiliations:** 1 Department of Chemistry “Ugo Schiff”, University of Florence, Via della Lastruccia 13, 50019 Sesto Fiorentino, Italy; 2 Interdepartmental Laboratory of Peptide and Protein Chemistry and Biology, Via della Lastruccia 13, 50019 Sesto Fiorentino, Italy; 3 Department of Chemical Sciences and Technologies, University of Rome ‘Tor Vergata’, Via Ricerca Scientifica 1, 00133 Rome, Italy; 4 Department of Neurosciences, Psychology, Drug Research and Child Health—Section of Pharmaceutical Sciences and Nutraceutics, University of Florence, Via Ugo Schiff 6, 50019 Sesto Fiorentino, Italy; 5 PeptLab@UCP Platform and Laboratory of Chemical Biology EA4505, University of Cergy-Pontoise, 5 mail Gay-Lussac, 95031 Cergy-Pontoise CEDEX, France; 6 Department of Chemistry, University of Padova, Via Marzolo 1, 35131 Padova, Italy

**Keywords:** self-assembled monolayers, tethered bilayer lipid membranes, electrochemical impedance spectroscopy, cyclic voltammetry, large unilamellar vesicles, fluorescence, multiple sclerosis, autoantibodies, *β*-turn peptide structures

## Abstract

The interaction of lipid environments with the type I’ *β*-turn peptide structure called CSF114 and its *N-*glucosylated form CSF114(Glc), previously developed as a synthetic antigenic probe recognizing specific autoantibodies in a subpopulation of multiple sclerosis patients’ serum, was investigated by fluorescence spectroscopy and electrochemical experiments using large unilamellar vesicles, mercury supported lipid self-assembled monolayers (SAMs) and tethered bilayer lipid membranes (tBLMs). The synthetic antigenic probe *N-*glucosylated peptide CSF114(Glc) and its unglucosylated form interact with the polar heads of lipid SAMs of dioleoylphosphatidylcholine at nonzero transmembrane potentials, probably establishing a dual electrostatic interaction of the trimethylammonium  and phosphate groups of the phosphatidylcholine polar head with the Glu^5^ and His^9^ residues on the opposite ends of the CSF114(Glc) *β*-turn encompassing residues 6-9. His^9^ protonation at pH 7 eliminates this dual interaction. CSF114(Glc) is adsorbed on top of SAMs of mixtures of dioleoylphosphatidylcholine with sphingomyelin, an important component of myelin, whose proteins are hypothesized to undergo an aberrant *N-*glucosylation triggering the autoimmune response. Incorporation of the type I’ β-turn peptide structure CSF114 into lipid SAMs by potential scans of electrochemical impedance spectroscopy induces defects causing a slight permeabilization toward cadmium ions. The *N-*glucopeptide CSF114(Glc) does not affect  tBLMs to a detectable extent.

## 1. Introduction

The type I’ *β*-turn glycopeptide CSF114(Glc) is an *N-*glucosylated antigenic probe specifically designed to recognize autoantibodies present in the sera of multiple sclerosis patients [1,2,3,4,5]. Its primary structure is TPRVERN(Glc)GHSVFLAPYGWMWK. At pH 3 the unglucosylated peptide CSF114 has a positive net charge of +4, due to a histidine, a lysine and two arginine residues. Its positive net charge decreases to +3 in a pH 5.4 unbuffered aqueous solution, following deprotonation of the glutamic acid residue, and it decreases further to +2 at pH 7, following deprotonation of the histidine residue. Its calculated structure includes two mini *β*-sheets, involving residues 4-5 and 10-11, and two turned motifs encompassing residues 6-9 and 14-17 [3].

In view of the relevance of the *N-*glucosylated peptide CSF114(Glc) in the identification of autoantibodies that were previously demonstrated as biomarkers of a subpopulation of multiple sclerosis patients [3,4], we found it interesting to verify to what extent this *N-*glucosylated peptide may interact with the lipid component of membranes. This paper aims at investigating the interactions of the unglucosylated type I’ *β*-turn peptide structure CSF114 and of its *N-*glucosylated form (the only one detecting antibodies in Multiple Sclerosis) by employing three different biomimetic membranes, *i.e.*, large unilamellar vesicles (LUVs), a mercury-supported lipid self-assembled monolayer (SAM) and a mercury-supported tethered bilayer lipid membrane (tBLM). These biomimetic membranes were immersed in aqueous 0.1 KCl at different pH values ranging from 3 to 7. The tBLM was obtained by tethering a thiolipid monolayer to the mercury surface. The thiolipid, called DPTL [6], consists of a tetraethyleneoxy hydrophilic chain, called spacer, terminated at one end with a lipoic acid residue for anchoring to the metal surface, and covalently linked at the other end to two phytanyl chains mimicking the hydrocarbon tails of a lipid. Self-assembling a phospholipid monolayer on top of the thiolipid monolayer gives rise to a lipid bilayer interposed between the aqueous solution and the hydrophilic spacer, which may accommodate a number of water molecules and ions, thus acting as an ionic reservoir [7]. It may accommodate up to two potassium ions per DPTL molecule, corresponding to a charge density of about 45 μC cm^−2^. This mercury-supported tBLM has been extensively employed in our laboratory for the investigation of ion channels [8,9,10,11,12,13,14,15,16,17].

A mercury-supported lipid SAM turns the hydrocarbon tails toward the hydrophobic mercury surface and the polar heads toward the aqueous solution [18]. Even though this biomimetic membrane consists of a single lipid monolayer, under certain conditions it can be used for investigating the properties of channel-forming peptides that may span exactly a lipid monolayer, having a length comparable with the monolayer thickness (~3.2 nm). Gramicidin [19,20], trichogin [21] and amphotericin [22,23] are peptides satisfying this requirement. The ability of short peptides to form pores or channels in the membrane can be tested by verifying whether they allow the electroreduction of inorganic cations such as Tl^+^ and Cd^2+^, which do not permeate lipid SAMs over their potential range of stability, but are electroreduced on bare mercury with amalgam formation over this range. In this case, mercury itself provides an unlimited ionic reservoir to the above electroactive ions, if they may translocate across the SAM via pore-forming peptides. The effect of small peptides on a lipid SAM may also provide useful information on their initial interaction with the outer leaflet of a biological membrane. 

## 2. Results and Discussion

### 2.1. Fluorescence Measurements on LUVs

In order to study the behavior of the type I *β*-turn peptide structure CSF114 in free standing bilayers and in the absence of a transmembrane potential, experiments on suspensions of LUVs were performed.

#### 2.1.1. Peptide-Membrane Association

The interaction of the peptide CSF114 with DOPC membranes was investigated at pH 3 and 5 by exploiting the intrinsic fluorescence of its tryptophan (Trp) residue. A fixed peptide concentration was titrated with increasing amounts of vesicles (up to a lipid concentration of about 150 μM), following both the position of the emission spectrum and fluorescence anisotropy. The emission spectrum is sensitive to the polarity of the environment and is, therefore, very different in water and in a lipid bilayer [24]. Fluorescence anisotropy is a measure of the rotational diffusion of the probe during its excited state lifetime, and increases dramatically in passing from a free peptide in solution to one bound to a liposome 100 nm in diameter (with high anisotropy values corresponding to slow motions). For instance, values of about 0.03 and 0.2 were previously reported for the free and membrane-bound state of a peptide similar in size to CSF114 [25]. 

**Figure 1:**
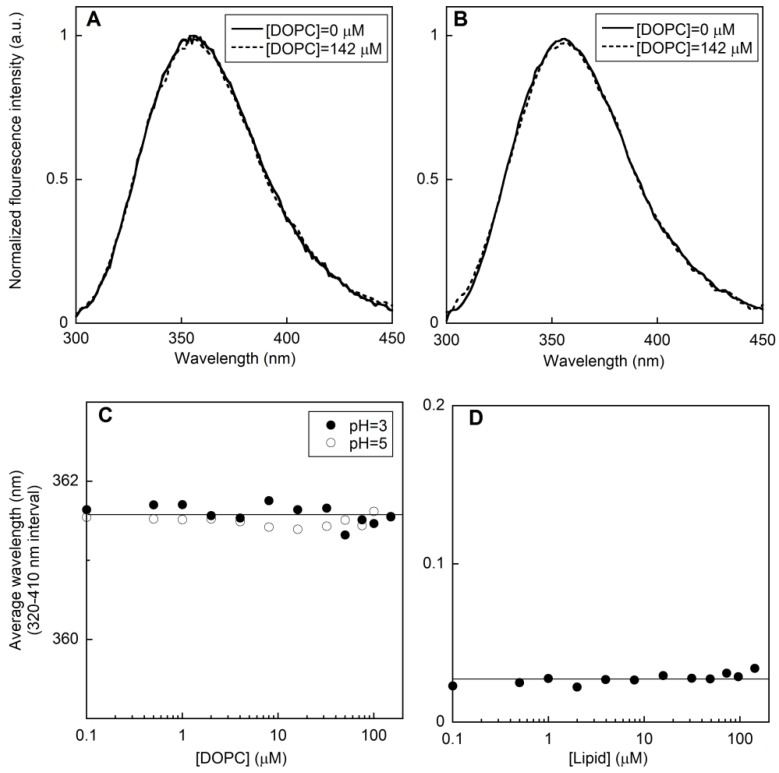
Normalized fluorescence intensity of 0.69 μM type I’ *β*-turn peptide structure CSF114 against wavelength in the absence (solid curve) and in the presence (dashed curve) of 142 μM DOPC at pH 3 (A) and pH 5 (B). Average wavelength against DOPC concentration at pH 3 and 5 (C). Fluorescence anisotropy of 2.0 μM CSF114 against lipid concentration at pH 3 (D).

At both pH values, no shift in the emission spectrum was observed (Figure 1A–C). This could indicate that under the employed experimental conditions the fraction of membrane-associated peptide is minor, if any, or that the peptide is associated to the bilayer in an orientation leaving its Trp residue exposed to the water phase. However, anisotropy experiments performed at pH 3 rule out the latter hypothesis, since no significant change was observed in the rotational mobility of the peptide (Figure 1D).

#### 2.1.2. Membrane perturbation induced by the type I’ *β*-turn peptide structure CSF114

**Figure 2:**
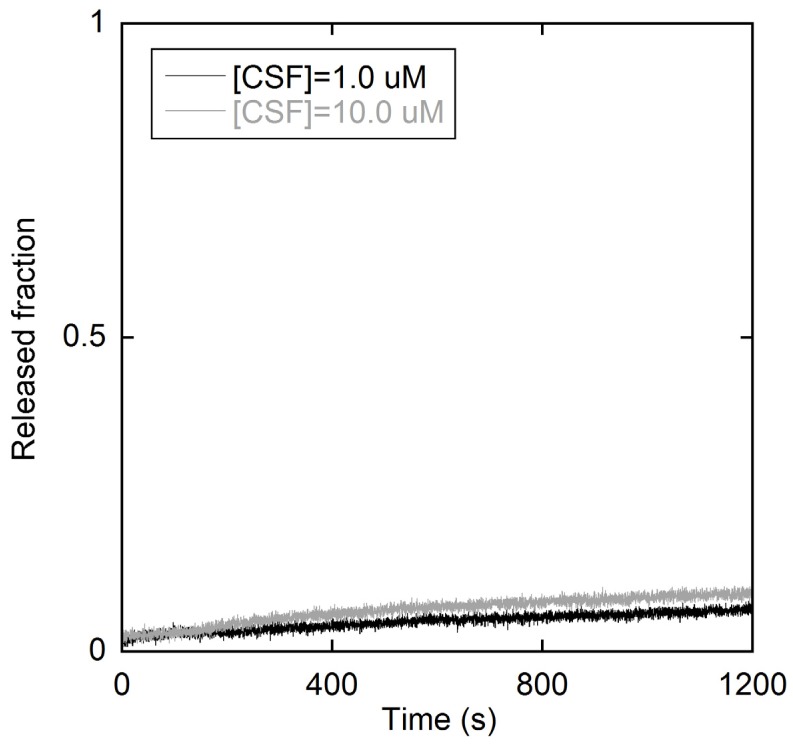
Kinetics of calcein release after addition of the type I’ *β*-turn peptide structure CSF114 to a LUV suspension. [Lipid] = 50 µM and [Peptide] = 1.0 µM and 10.0 µM.

Calcein-loaded liposomes were used to assess the membrane-perturbing activity of the peptide CSF114. The dye was entrapped in the vesicles at a self-quenching concentration, so that peptide-induced release would cause a dilution in the outer solution and an increase in fluorescence intensity [26]. The leaked fraction *R* was calculated as a function of time *t* after peptide addition, according to the equation: *R*(*t*) = (*F*(*t*)–*F*_0_)/(*F*_100_–*F*_0_). Here, *F*_0_ is the calcein signal before peptide addition and *F*_100_ is the intensity after the addition of a detergent (1 mM triton-X100), which causes complete lysis of membranes. *F*(*t*) is the fluorescence value obtained at time *t*. Only a slight leakage (less than 10%) was observed in 20 minutes, even after increasing the peptide concentration to 10 µM (Figure 2).

Summarizing, the experiments performed on LUVs, in the absence of a transmembrane potential, show a negligible binding of the peptide structure to membranes and a marginal peptide-induced bilayer leakage, at both pH 3 and 5. These findings suggest that a transmembrane potential is essential for both the association of type I’ *β*-turn peptide structure CSF114 to membranes and its membrane-perturbing activity.

### 2.2. Electrochemical Measurements at Mercury-Supported Lipid SAMs and tBLMs

All measurements at phospholipid SAMs and tBLMs were carried out with both the unglucosylated form of the type I’ *β*-turn peptide structure CSF114 and the N-glucosylated form, CSF114(Glc). No significant differences in the effect of the two different forms were detected. Hence, the following results refer to measurements performed with the unglucosylated peptide structure CSF114.

#### 2.2.1. Interaction of the type I’ *β*-turn peptide structure CSF114 with Hg-supported DOPC and DOPS SAMs

**Figure 3:**
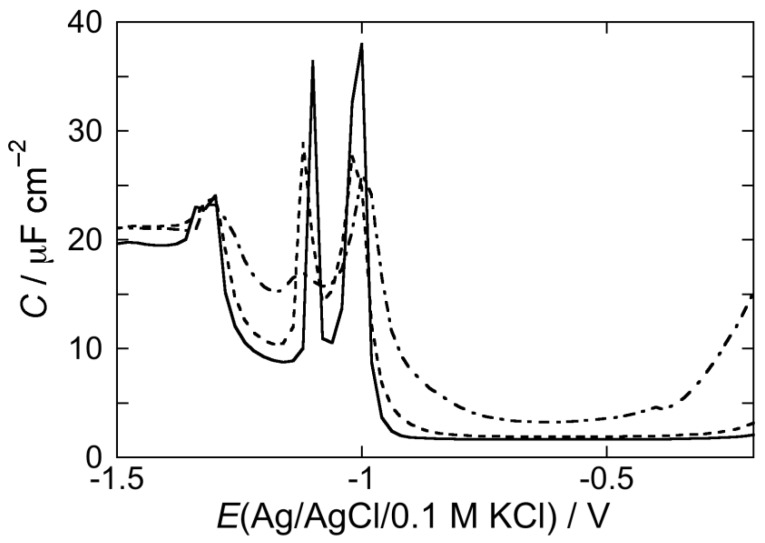
AC voltammogram at a DOPC SAM in a pH 3 solution of 0.1 M KCl in the absence of the type I’ *β*-turn peptide structure CSF114 (solid curve), and in the presence of 2 μg/mL CSF114 after five potential scans from –0.2 to –1.5 V (dashed curve) and after a single EIS scan (dash-dotted curve). Frequency = 75 Hz.

Figure 3 shows the AC voltammogram of a DOPC SAM in a pH 3 aqueous solution of 0.1 M KCl, in which the capacitance *C* is plotted against the applied potential *E*. Over the potential region of minimum capacitance, which ranges from −0.20 to −0.80 V, the DOPC monolayer is impermeable to inorganic ions, whereas it becomes permeable outside this region. The *C* value over this region amounts to 1.8 μF cm^−2^, namely twice the value for a solvent-free BLM [18]. At positive potentials the region of minimum capacitance is delimited by a capacitance increase that precedes mercury oxidation. At negative potentials it is delimited by a sharp pseudo-capacitance peak that lies at about −1.02 V, followed by two further peaks at about −1.10 V and −1.35 V. The first two peaks are ascribed to a cooperative reorientation of the lipid molecules, whereas the third one is due to their partial desorption [27]. The first peak results from surface defects that allow a practically uninhibited access of inorganic ions to the mercury surface, whereas the second peak results from nucleation and growth of the defects formed during the first peak, causing their coalescence. Fluorescence measurements show that the lipid molecules desorbed at potentials sufficiently negative with respect to the third peak form aggregates far enough from the mercury surface to prevent the quenching of fluorophores mixed with the lipid molecules [28]. These aggregates do not migrate about, but spread again on the mercury surface at less negative potentials, reconstituting a lipid film. 

As a rule, molecules capable of penetrating the hydrocarbon tail region of the phospholipid monolayer increase its capacitance over the potential range of the flat capacitance minimum with respect to its value, 1.8 μC cm^–2^, in the absence of foreign species, if their polarizability is appreciably higher than that of the lipid molecules. Conversely, they affect the monolayer capacitance only slightly if they have a low polarizability [29]. In both cases, if their concentration in the lipid monolayer is sufficiently high, their intercalation with the lipid molecules prevents the latter molecules from undergoing a sufficiently sharp cooperative reorientation, thus broadening and depressing the two pseudo-capacitance peaks in the solid curve of Figure 1. Molecules adsorbed on top of the lipid monolayer, but unable to penetrate it, interact with the polar heads along the whole stability range of the lipid SAM. However, such an interaction is usually not detected along the region of the flat capacitance minimum; it is only monitored at applied potentials negative enough to induce a somewhat disordered and non-cooperative reorientation of the lipid molecules, resulting in depressed pseudocapacitance peaks [22,30]. They may succeed in decreasing the capacitance over the potential range of the flat capacitance minimum only if they form a sufficiently compact layer, thus increasing the thickness of the whole adsorbed material [29]. 

In the absence of exogenous species, scanning the potential from the region of the flat capacitance minimum to a negative value lying between the second and third peak in Figure 3, and then scanning it backwards, restores the original capacitance and lipid ordered arrangement. However, in the presence of exogenous molecules, such a potential scan may favor their intercalation with the hydrocarbon tails and/or the polar heads of the lipid SAM over the region of the flat capacitance minimum, provided they have some affinity for it. Another procedure adopted to favor the possible penetration of an exogenous molecule consists in carrying out a series of electrochemical impedance spectra, at regular intervals of the bias potential, over the potential range of stability of the SAM. This procedure will be briefly referred to as an ‘EIS scan’. In this case, incorporation may be favored by the relatively long time during which the SAM is maintained over its stability range and/or by some effect of the small AC signals covering the frequency range from 10^–1^ to 10^5^ Hz.

Before considering the interaction of CSF114 with mercury-supported DOPC and DOPS monolayers, it is convenient to briefly examine how a change in the applied potential *E* is distributed over the hydrocarbon tail and the polar head region. Under the present experimental conditions, the further potential difference distributed across the diffuse layer region adjacent to lipid SAM is negligible, in view the low change density *σ*_M_ on the mercury surface [27] and to the relatively high 0.1 M KCl electrolyte concentration adopted in all measurements. The polar head region is also the site of a surface dipole potential Δ*χ*, estimated at about 200 mV, positive toward the hydrocarbon tail region [31]; since it does not vary with the applied potential *E*, it can be disregarded in all measurements dealing with the effect of changes in *E*. In general, the potential differences across the hydrocarbon tail and polar head regions are given by *βσ*_M_/(*ε*_0_*ε_β_*) and *γ*(*σ*_M_+*σ*_i_)/(*ε*_0_*ε*_γ_), respectively, where *β* and *γ* are the thicknesses of the hydrocarbon tail and polar head regions, *ε_β_* and *ε*_γ_ are the corresponding dielectric constants, *ε*_0_ is the permittivity of free space, *σ*_M_ is the charge density on the mercury surface and *σ*_i_ is the charge density of any ionized group buried well inside the polar head region [32]. In practice, *σ*_i_ is different from zero and negative only at a DOPS monolayer, when its phosphate group, which is embedded in the polar head region in its most common conformation, is deprotonated. In practice, due to its embedded location, the phosphate group is protonated at pH values < 6 [33]. In what follows, we will set *σ*_i_ = 0 for simplicity, disregarding the possible negative charge of the DOPS phosphate group at pH 7. The space-filling model of a DOPC or DOPS molecule yields a *β* value of about 0.8 nm and a *γ* value of about 0.5 nm, if the ester linkages of the two oleic acid residues with the glycerol backbone are included in the polar head region. The differential capacitance *C* of the whole phospholipid monolayer in the absence of exogenous species is about equal to 1.8 μF cm^–2^, and its reciprocal is clearly equal to the sum of the reciprocals of the differential capacitances, *ε*_0_*ε_β_*/*β* and *ε*_0_*ε*_γ_/*γ*, of the hydrocarbon tail and polar head regions. The dielectric constant *ε*_γ_, as obtained from the above relationship upon setting *ε_β_*= 2, amounts to 5.5. The charge density *σ*_M_ on a DOPC-coated mercury drop estimated from chronocoulometric measurements attains its zero value at about –20 mV *vs.* the Ag/AgCl/(0.1 M KCl) reference electrode [27,34]. Noting that the C value along the whole flat capacitance region of the AC voltammogram is practically constant and equal to 1.8 μF cm^–2^, the *σ*_M_ value along this region can be approximately equated to *CE*. Consequently, the potential differences across the hydrocarbon tail and polar head regions are given by:

(1) $$\frac{\beta \sigma_{M}}{\varepsilon_{0}\varepsilon_{\beta }}=\frac{\beta CE}{\varepsilon_{0}\varepsilon_{\beta }}=\frac{\beta /\varepsilon_{\beta }}{\beta /\varepsilon_{\beta }+\gamma /\varepsilon_{\gamma }}E;\frac{\gamma \sigma_{M}}{\varepsilon_{0}\varepsilon_{\gamma }}=\frac{\gamma CE}{\varepsilon_{0}\varepsilon_{\gamma }}=\frac{\gamma /\varepsilon_{\gamma }}{\beta /\varepsilon_{\beta }+\gamma /\varepsilon_{\gamma }}E$$

Replacing the appropriate numerical values into Equation (1), the potential differences across these two regions are given by 0.81 *E* and 0.19 *E* throughout the whole flat capacitance region. It is evident that the potential difference across the polar head region cannot be disregarded with respect to that across the hydrocarbon tail region. Equation (1) holds strictly at constant applied potential *E*. As *E* is varied following an AC signal or a voltammetric voltage scan, the resulting transient change in the slight ionic charge density located within the polar head region of the lipid SAM may produce a modest change in the potential differences across the hydrocarbon tail and polar head regions with respect to those expressed by this equation. The potential differences across the head groups and the hydrocarbon tails depend on the assumed values of dielectric constant and charge density to a large extent. They are not only as expressed by their average values, but are also influenced by transient effects, and care must be taken to avoid significant variations of the potential throughout the chosen scan time.

A roughly approximate expression for the extrathermodynamic potential difference Δ*φ* across the whole phospholipid SAM must also include the surface dipole potential Δ*χ*:

(2) $$\Delta \chi =\frac{\beta /\varepsilon_{\beta }}{\beta /\varepsilon_{\beta }+\gamma /\varepsilon_{\gamma }}E+\frac{\gamma /\varepsilon_{\gamma }}{\beta /\varepsilon_{\beta }+\gamma /\varepsilon_{\gamma }}E+\Delta \chi ≅E+0.200V$$

Hence, the absolute potential difference Δ*φ* across a mercury-supported lipid monolayer, which can be viewed as a “transmembrane potential”, is roughly estimated by increasing the potential difference measured *vs.* the Ag/AgCl/(0.1 M KCl) reference electrode by about 200 mV. The approximate expression of Equation (2) is similar to a more general expression relying on more accurate extrathermodynamic considerations [35]. It serves to show that the absolute potential difference across a mercury-supported phospholipid monolayer, which is equivalent to a “transmembrane potential”, falls within the range of physiological values over the more positive portion of the region of minimum capacitance.

Figure 3 shows AC voltammograms at a DOPC SAM in a pH 3 solution of 0.1 M KCl. The solid curve was obtained in the absence of the type I’ *β*-turn peptide structure CSF114, whereas the dashed curve was obtained in the presence of 2 μg/mL CSF114 after scanning the potential five times from −0.2 to −1.5 V. Coincidence of the AC voltammogram in the presence of CSF114 with that in its absence in the middle of the flat capacitance minimum, where the SAM shows its maximum stability, denotes the absence of peptide penetration there. The increase in capacitance on the two sides of the capacitance minimum, where the SAM stability is lower, denotes some penetration of CSF114 in the hydrocarbon tail region. Depression of the first peak and complete suppression of the second are indicative of an interaction of the type I’ *β*-turn peptide structure with the polar heads of the DOPC SAM. The dash-dotted curve in Figure 3 was obtained after an EIS scan. It is evident that this procedure induces some penetration of CSF114 over the whole stability range of the SAM.

**Figure 4:**
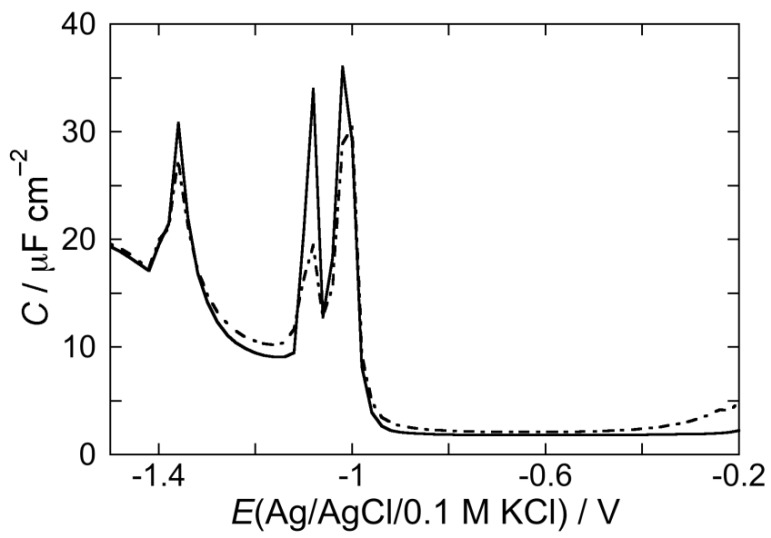
AC voltammogram at a DOPC SAM in a pH 7 buffer solution of 0.1 M in the absence of the type I’ *β*-turn peptide structure CSF114 (solid curve), and in the presence of 2 μg/mL CSF114 after a single EIS scan (dash-dotted curve). Frequency = 75 Hz.

The AC voltammetry curves in a pH 5.4 unbuffered solution behave similarly to those at pH 3, albeit with a slightly lower effect of CSF114. Conversely, in a pH 7 phosphate buffer solution, addition of 2 μg/mL CSF114 has no effect after five potential scans from −0.2 to −1.5 V and a very small effect after an EIS scan, as shown by the dash-dotted curve in Figure 4.

**Figure 5:**
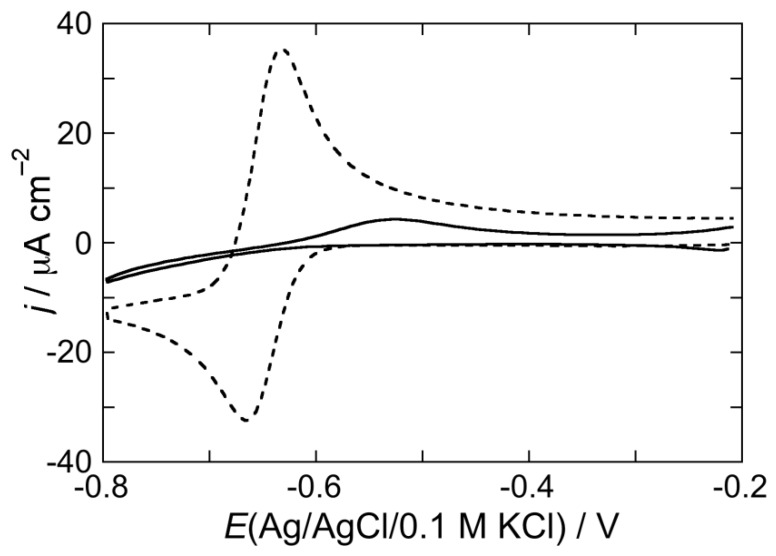
Cyclic voltammogram at a DOPC SAM in a pH 5.4 unbuffered solution of 0.1 M KCl, 4 × 10^−5^ M CdSO_4_ and 2 μg/mL of the type I’ *β*-turn peptide structure CSF114 (solid curve). The dashed curve is the cyclic voltammogram at bare mercury in an unbuffered solution of 0.1 M KCl and 4 × 10^−5^ M CdSO_4_. Scan rate = 0.05 V/s.

To verify whether the increase in capacitance over the whole potential range of stability of the DOPC SAM, induced by CSF114 at pH 3 and 5.4 after an EIS scan, may be associated to SAM permeabilization toward inorganic ions, cyclic voltammograms over the same potential range were recorded both in the absence and in the presence of the electroactive Cd^2+^ ion. The cyclic voltammogram due to Cd^2+^ electroreduction to cadmium amalgam, Cd(Hg), and to the reverse electrode reaction at a DOPC SAM bathed by a pH 5.4 unbuffered solution of 0.1 M KCl, 4 × 10^−5^ M CdSO_4_ and 2 μg/mL CSF114 is very low (solid curve in Figure 5) with respect to that at a bare mercury electrode (dashed curve in Figure 5), with that at pH 3 being slightly higher than that at pH 5.4. This behavior indicates that the type I’ *β*-turn peptide structure CSF114 penetrates only in the polar head region of the DOPC SAM and does not span the whole lipid monolayer. If the peptide spanned the membrane, cadmium ions would permeate it and be electroreduced to cadmium amalgam to an appreciable extent.

We have recently verified that strong interactions with the polar heads of a DOPC SAM are established by peptides that have at least a pair of contiguous oppositely charged residues in their peptide chain. This behavior has been explained by a dual electrostatic interaction of the negative residue with the trimethylammonium group of a DOPC polar head and of the contiguous positive residue with the phosphate group of the same polar head. Thus, e.g., the dermcidin antimicrobial peptide establishes strong interactions with the polar heads of a DOPC SAM in a pH 5.4 unbuffered solution of 0.1 M KCl thanks to the presence of three pairs of contiguous oppositely charged residues, namely Glu^5^-Lys^6^, Lys^23^-Asp^24^ and Lys^41^-Asp^42^ [15]. An example more relevant to the present work is provided by the small protein sarcolipin, which modulates the function of Ca^2+^-ATPase of the sarcoplasmic reticulum. Sarcolipin has a positive arginine residue (Arg^6^) and a contiguous glutamic residue (Glu^7^) in its cytoplasmic domain; it suppresses the pseudo-capacitance peaks in the AC voltammogram of the DOPC SAM at pH 7, but depresses them only slightly in a pH 5.4 unbuffered solution [34]. This behavior was explained by Glu^7^ being deprotonated, and hence negatively charged, at pH 7, but protonated, and hence neutral, in unbuffered solution. In fact, even though the pK_a_ value of glutamic acid is usually close to 4.2, it may be appreciably increased when the amino acid is inside the peptide sequence, often assuming values ranging from 6 to 6.5. It is, therefore, surprising that CSF114, which has an identical pair of contiguous oppositely charged residues, namely Glu^5^ and Arg^6^, exerts an opposite effect on the DOPC SAM, by suppressing its pseudo-capacitance peaks at pH 5.4 and affecting them only slightly at pH 7. This behavior can be reasonably explained by assuming that a dual electrostatic interaction with the trimethylammonium and phosphate groups of a DOPC polar head is established by the Glu^5^ and His^9^ residues, which are at the opposite ends of the *β*-turn encompassing residues 6-9 in the lowest energy CSF114 conformer. The *β*-turn may bring the glutamic and histidine residues close to each other, favoring their dual interaction with a DOPC polar head. Since histidine has a pK of 6.04, it is expected to be mainly positively charged at pH 5.4 and mainly neutral at pH 7. Hence, at pH 5.4 it establishes a particularly strong interaction with a DOPC phosphate. On the other hand, in a pH 7 phosphate buffer, not only are the histidine residues mainly in the neutral deprotonated form, but also the remaining protonated residues tend to escape interactions with the DOPC SAM by interacting with the phosphate ions of the buffer.

**Figure 6:**
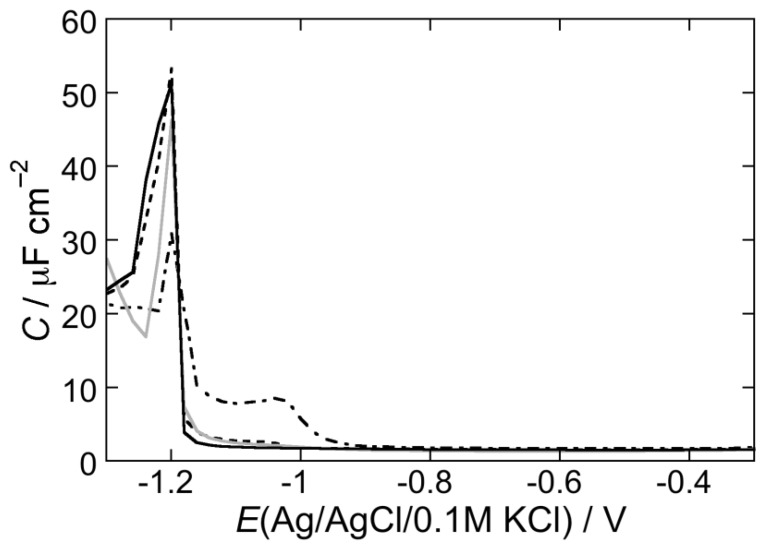
AC voltammogram at a DOPC SAM in a pH 3 aqueous solution of 0.1 M KCl in the absence of the type I’ *β*-turn peptide structure CSF114 (solid curve), and in the presence of 2 μg/mL CSF114 after five potential scans from −0.2 to −1.5 V (dashed curve) and after a single EIS scan (dash-dotted curve). The gray curve is the AC voltammogram at a DOPC SAM in a pH 7 buffer solution of 0.1 M KCl and 2 μg/mL CSF114 after a single EIS scan. Frequency = 75 Hz.

Figure 6 shows the AC voltammogram of a DOPS SAM bathed by a pH 3 aqueous solution of 0.1 M KCl in the absence of CSF114 (solid curve), in the presence of 2 μg/L CSF114 after five voltage scans (dashed curve), and after an EIS scan (dash-dotted curve). Incidentally, the sole DOPS monolayer (solid curve) was shown to be positively charged at pH 3, almost neutral around pH 6 and negatively charged with a further increase in pH [33]. This behavior was tentatively explained by a conformation with the carboxyl and the amino groups of adjacent DOPS molecules coplanar to the monolayer and characterized by electrostatic dipole-dipole interactions; the phosphate group, with a pK of about 8, is buried inside the polar head region and is almost completely protonated at pH 6. In the presence of 2 μg/mL CSF114, five voltage scans have practically no effect, whereas the EIS scan increases the capacitance only at potentials negative of −0.90 V, where the stability of the monolayer decreases, and depresses the pseudo-capacitance peak. An identical behavior is shown at pH 5.4. In a pH 7 phosphate buffer, the curve of the differential capacitance against potential is only very slightly affected even after an EIS scan (gray curve in Figure 6).

The chameleonic adaptability of the DOPS conformation to even slightly different environmental conditions is testified by the notable scatter in the pK values of its carboxyl and phosphate groups reported in the literature [33]. The protonated state of the phosphate groups of DOPS is endangered by any exogenous species capable of disrupting the planar arrangement of the carboxyl-amino dipoles between adjacent DOPS molecules. Such an effect is possibly exerted by the positively charged side chains of CSF114, which may shift the equilibrium from the protonated to the deprotonated form of the phosphate group by forming H-bonds with the latter. This CSF114 effect on the DOPS monolayer is exclusively limited to a depression of its pseudo-capacitance peak, while the region of the flat capacitance minimum is left unaltered. A mayor role played by the histidine residue on H-bond formation, with respect to the lysine or arginine residues, is suggested by the negligible effect exerted by the type I’ *β*-turn peptide structure CSF114 on the pseudo-capacitance peak in the pH 7 phosphate buffer, when this residue is mainly in the neutral deprotonated form. Moreover, the positively charged residues are expected to form H-bonds with the phosphate ions of the pH 7 buffer, deviating them from H-bonding to DOPS.

**Figure 7:**
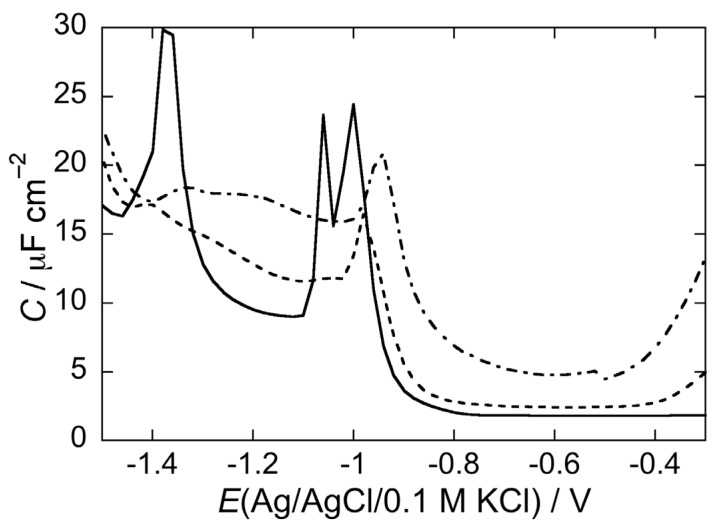
AC voltammogram at a SAM of a DOPC/PSM (2:1) mol% lipid mixture in a pH 5.4 unbuffered solution of 0.1 M KCl in the absence of CSF114 (solid curve), and in the presence of 2 μg/mL CSF114 after five potential scans from −0.2 to −1.5 V (dashed curve) and after a single EIS scan (dash-dotted curve). Frequency = 75 Hz.

The structure-based designed *N-*glucosylated peptide CSF114(Glc) [3] was demonstrated to be able to identify autoantibodies in the sera of a subpopulation of multiple sclerosis patients [4] by a simple immunoenzymatic assay [36] but also with SPR-based techniques [37,38]. Antibody titre correlated with disease activity [4]. The glycopeptide is characterized by a type I’ *β*-turn structure bearing, as minimal epitope, a *β*-D-glucopyranosyl moiety linked by an amide bond to an Asn residue on the tip of the turn [42,43], possibly reproducing an aberrant *N-*glucosylation of myelin proteins fundamental for autoantibody recognition [43,44]. This aberrant *N-*glucosylation of myelin proteins may generate neoantigens no longer recognized as self, thus triggering the autoimmune response [42,43]. Therefore, it was found of interest to investigate the effect of the type I’ *β*-turn CSF114 and CSF114(Glc) at a bicomponent SAM formed by a DOPC/palmitoylsphingomyelin (PSM) (2:1) mol% lipid mixture, in view of the fact that sphingomyelin is an important component of the myelin sheath. Both the unglucosylated CSF114 and the *N-*glucosylated CSF114(Glc) peptides are rapidly and effectively incorporated in this SAM after five voltage scans, causing a capacitance increase along the flat capacitance minimum and a depression of the two pseudo-capacitance peaks of the DOPC/PSM mixed monolayer, as shown by the dashed curve in Figure 7, which refers to CSF114. This effect is more pronounced after an EIS scan than after five potential scans. 

**Figure 8:**
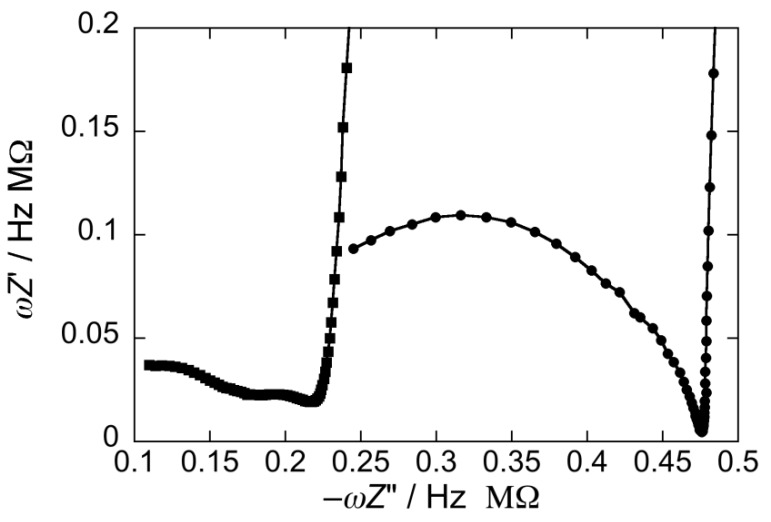
Plot of *ωZ*’ against –*ωZ*” at –0.40 V for a SAM of a DOPC/PSM (2:1) mol% lipid mixture in a pH 5.4 unbuffered solution of 0.1 M KCl in the absence of CSF114 (solid circles) and in the presence of 2 μg/mL CSF114 (solid squares).

The electrochemical impedance spectrum of this system, when displayed on a plot of *ω*Z’ against −*ω*Z” (M plot), exhibits a single distorted semicircle in the absence of CSF114, as shown at −0.40 V by the solid circles in Figure 8. *Z*’ and *Z*” denote the in-phase and quadrature component of the impedance, and *ω* is the angular frequency. Addition of 2 μg/mL CSF114 generates an additional small semicircle (solid squares in Figure 8), falling at higher frequencies with respect to the former one. It should be noted that an adsorbed film can be regarded as consisting of a series of different slabs with distinct dielectric properties [44]. Each slab is simulated by a parallel combination of a resistance *R* and a capacitance *C*, namely a ‘RC mesh’. On an M plot, a single RC mesh yields a semicircle whose diameter equals the reciprocal of its capacitance. Moreover, the angular frequency *ω* at the maximum of each semicircle is equal to the reciprocal of the time constant, *RC*, of the corresponding mesh. The second semicircle in Figure 8 (solid squares) has a much smaller diameter than the first one, ascribable to the DOPC monolayer, and hence a much higher capacitance. Noting that *ω* increases along the positive direction of the −*Z*”*ω* axis, the second semicircle has a time constant lower than that of the first one, and hence a lower resistance, which more than counterbalances its higher capacitance.

**Figure 9:**
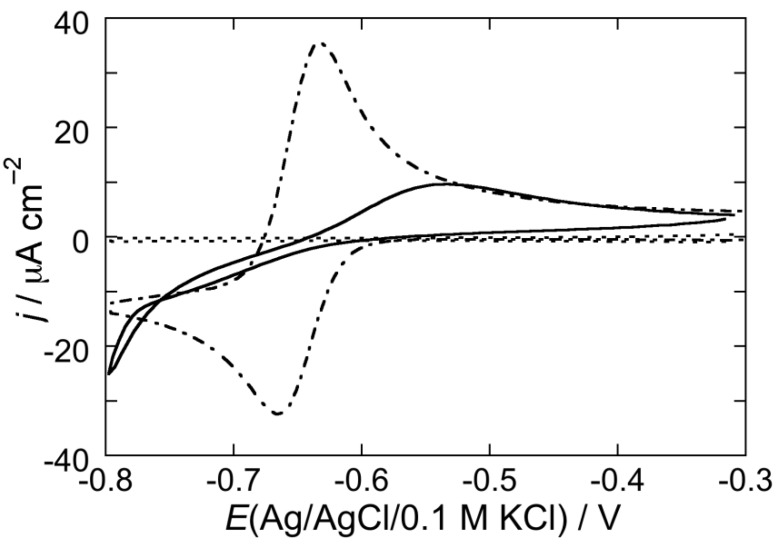
Cyclic voltammogram at a SAM of a DOPC/PSM (2:1) mol% lipid mixture in a pH 5.4 unbuffered solution of 0.1 M KCl and 4 × 10^−5^ M CdSO_4_ in the absence of CSF114 (dashed curve) and in the presence of 2 μg/mL CSF114 (solid curve). The dash-dotted curve is the cyclic voltammogram on bare mercury in unbuffered solution of 0.1 M KCl and 4 × 10^−5^ M CdSO_4_. Scan rate = 0.05 V/s.

CSF114 permeabilizes the DOPC/PSM SAM towards Cd^2+^ ions to an appreciable extent, as appears from Figure 9, where the cyclic voltammogram of 4 × 10^−5^ M CdSO_4_ at the SAM in a pH 5.4 unbuffered solution of 0.1 M KCl and 2 μg/mL CSF114 (solid curve) is compared with that at bare mercury (dash-dotted curve), under otherwise identical conditions. The dotted curve is the cyclic voltammogram in the absence of CSF114.

The above behavior of the DOPC/PSM SAM can be explained by considering that it consists of PSM-rich gel phase microdomains surrounded by a DOPC-rich “liquid-disordered” matrix [45,46]. Peripheral peptides, such as the type I’ *β*-turn CSF114, tend to accumulate on top of gel phase microdomains, generating an additional slab of low resistance and high capacitance, which is responsible for the additional small semicircle in Figure 8. In addition, the anisotropy of gel phase microdomains determines a mismatch along their boundary with the matrix, where inorganic ions can more easily penetrate the SAM [47,48]. The back and forth movement of electro-inactive ions under an AC voltage determines an increase in differential capacitance. Moreover, Cd^2+^ ions can more easily reach the mercury surface and be electroreduced with amalgam formation. 

A lipid composition that approaches more closely that of the myelin sheath also includes cholesterol (Chol), an essential constituent of myelin. To this end, the effect of CSF114 was tested at a DOPC/Chol/PSM SAM of (50:30:20) mol% composition, in a pH 5.4 unbuffered solution of 0.1 M KCl. According to the phase diagram of the POPC/Chol/PSM lipid mixture estimated by de Almeida *et al.* [49,50], where POPC stands for palmitoyloleoylphosphatidylcholine, this composition is characterized by the presence of isotropic microdomains rich in PSM and Chol, called lipid rafts, surrounded by a POPC-rich liquid disordered matrix. This result can be safely extended to the DOPC/Chol/PSM (50:30:20) mixture, since the two low-*T*_m_ components DOPC and POPC are expected to exhibit practically the same behavior. The impedance spectra of this lipid mixture, both in the absence and in the presence of 2 μg/mL CSF114, were fitted by an equivalent circuit consisting of a series of three RC meshes. The three RC meshes are meant to simulate the hydrocarbon tail region of the lipid SAM, its polar head region and the aqueous phase bathing the SAM. The peptide was found to affect the hydrocarbon tail region only slightly, causing its capacitance to remain substantially constant at 3.9 ± 0.1 μF cm^–2^ and decreasing its resistance from 0.4 to 0.2 MΩ cm^2^. Conversely, it decreases the resistance of the polar head region from 20 to 4 ± 1 kΩ cm^2^, while leaving the corresponding capacitance almost unaltered at about 5 μF cm^–2^. Lipid rafts are considered to act as platforms for the preferential sorting of peripheral proteins and peptides [51]. Therefore, the type I’ *β*-turn peptide CSF114 is expected to be preferentially adsorbed on rafts, which, being isotropic, do not determine mismatches at their boundaries. This explains why the differential capacitance remains substantially unchanged after peptide addition. 

#### 2.2.2. Interaction of the type I’ *β*-turn peptide CSF114 with Hg-supported DPTL/DOPC and DPTL/DOPS tBLMs

Mercury-supported tBLMs differ from phospholipid SAMs not only by their bilayer structure, but also by the presence of a hydrophilic tetraethyleneoxy chain (called spacer, since it is interposed between the mercury surface and the lipid bilayer), which may accommodate inorganic ions. If an exogenous species is capable of forming pores or ion channels in the lipid bilayer moiety of the tBLM, the resulting ion flow into the spacer is easily monitored by the use of various electrochemical techniques. In fact, an inflow of cations is accompanied by a simultaneous flow of electrons to the mercury surface along the external circuit. The negative electron charge is practically equal in magnitude and opposite in sign to that of inflowing cations, in order to maintain the electroneutrality of the whole electrified interphase. The proximal leaflet of the lipid bilayer moiety of a tBLM consists of phytanyl chains bound by ether linkages to the tetraethyleneoxy spacer and, hence, lacks the polar head region. Conversely, the distal leaflet consists of a phospholipid monolayer self-assembled on top of the DPTL thiolipid monolayer tethered to the mercury surface. In the present work, DOPC and DOPS distal monolayers were employed. 

The CSF114 peptide has practically no effect on the above tBLMs bathed by aqueous 0.1 M KCl at pH 5.4 and 7, when covering the potential range from −0.30 to −1.00 V. A modest permeabilizing effect observed at pH 3 by covering the same potential range is ascribable to a decrease in the compactness of the lipid bilayer, when subjected to potentials negative of −0.70 V at this low pH value. 

## 3. Experimental Section 

Water was obtained by an inverted osmosis unit; it was then distilled once and redistilled from alkaline permanganate. Merck (Darmstadt, Germany) suprapur^®^ KCl was baked at 500 ºC before use to remove any organic impurities. TlNO_3_, CdSO_4_, HCl and K_2_HPO_4_ from Merck were used without further purification. 2,3,di-O-phytanyl-sn-glycerol-1-tetraethylene-glycol-D,L-*α* lipoic acid ester lipid (DPTL) was provided by Prof. Adrian Schwan (Department of Chemistry, University of Guelph, Canada). Solutions of 0.2 mg/mL DPTL in ethanol were prepared from a 2 mg/mL solution of DPTL in ethanol. Stock solutions of this thiolipid were stored at −18 ºC. CSF114 and CSF114(Glc) peptide chains were independently synthesized in solid phase by Fmoc/tBu strategy and purified by RP-HPLC as previously described to a final purity >95% [52]. Dioleoylphosphatidylcholine (DOPC) and dioleoylphosphatidylserine (DOPS) for measurements at Hg-supported lipid tBLMs and SAMs were purchased in chloroform solution from Avanti Polar Lipids (Birmingham, AL, USA). DOPC, calcein, Triton X-100, Sephadex G-50, buffers and salts for measurements at LUVs were purchased from Sigma (St. Louis, MO, USA).

### 3.1. Liposome Preparation

Large unilamellar vesicles (LUVs) were prepared as previously described [53], by dissolving DOPC in a 1:1 (vol/vol) methanol/chloroform solution. The solvents were evaporated in a rotary evaporator until a thin film was formed. Complete evaporation was ensured by applying a rotary vacuum pump for at least 2 h. The lipid film was hydrated with a pH 3.0 100 mM KCl solution (for partition experiments at pH 3), with a pH 5.0 10 mM acetate buffer, 140 mM NaCl and 0.1 mM EDTA (for partition experiments at pH 5) or with a 30 mM calcein solution 10 mM phosphate buffer (pH 7.4), 50 mM NaCl and 0.1 mM EDTA (for membrane perturbation experiments). After vigorous stirring and 10 freeze and thaw cycles, the liposome suspensions were extruded for 31 times through two stacked polycarbonate membranes (Avestin, Ottawa, ON, Canada) with pores of 100 nm diameter. Liposomes prepared in the calcein solution were separated from the non-encapsulated fluorescent tracer by gel filtration on a Sephadex G-50 medium column (40 cm) in the pH 5.0 buffer solution. Lipid concentration in the final sample was determined by the Stewart method. 

### 3.2. Fluorescence Measurements

Fluorescence measurements were performed with a FluoroMax-4 fluorimeter (Horiba, Edison, NJ, USA). Peptide fluorescence was excited at 280 nm and spectra were collected with an integration time of 1 s and bandwidths of 2 nm and 3 nm in excitation and emission, respectively, while anisotropy was measured at an emission wavelength of 350 nm with a bandwidth of 7 nm both in excitation and emission, with an integration time of 15 s for each polarization. Calcein fluorescence was recorded with λ_exc_ = 490 nm, λ_em_ = 520 nm, bandwidths of 0.2 nm and 7 nm in excitation and emission, respectively, and an integration time of 0.2 s. The temperature was controlled at 25 °C for all experiments. The average spectral wavelength <λ> was calculated according to the equation: <*λ*> = (Σ_i_ *λ*_i_*I*_i_) / (Σ_i_*I*_i_), where *λ*_i_ represents each wavelength of the emission spectrum and *I*_i_ is the corresponding intensity.

### 3.3. Electrochemical Measurements

All measurements were carried out with a homemade hanging mercury drop electrode (HMDE) described elsewhere [54]. A homemade glass capillary with a finely tapered tip, about 1 mm in outer diameter, was employed. Capillary and mercury reservoir were thermostated at 25 ± 0.1 ºC in a water-jacketed box to avoid any changes in drop area due to a change in temperature. The HMDE acted as the working electrode in a three-electrode system, with an Ag/AgCl (0.1M KCl) reference electrode and a platinum coil counter electrode. Potentials are referred to the above reference electrode. Mercury-supported lipid monolayers were obtained by spreading a lipid solution in pentane on the surface of a buffered or unbuffered 0.1 M KCl aqueous solution, in an amount corresponding to about five phospholipid monolayers. After allowing the pentane to evaporate, the HMDE was immersed into the aqueous solution across the lipid film. This procedure gives rise to a lipid monolayer with the hydrocarbon tails directed toward the mercury surface and the polar heads directed toward the aqueous solution, thanks to the hydrophobic nature of the mercury surface. The lipid monolayer is at its equilibrium spreading pressure [27,28]. Mercury-supported tBLMs were obtained by tethering a DPTL monolayer on the HMDE upon keeping the mercury drop immersed in a 0.2 mg/mL DPTL solution in ethanol for about 20 min [9,10]. A DOPC monolayer was then formed on top of the DPTL monolayer by a procedure analogous to that employed for the preparation of mercury-supported lipid monolayers, and consisted of spreading a lipid solution in pentane on the surface of the working aqueous solution. Immersing the DPTL-coated mercury into the aqueous solution across the lipid film causes a lipid monolayer to self-assemble on top of the DPTL monolayer, thanks to the hydrophobic interactions between the alkyl chains of the phospholipid and those of the thiolipid [9]. The tBLM was then subjected to repeated potential scans over a potential range from −0.15 V to −1.15 V, while continuously monitoring the curve of the quadrature component of the current at 75 Hz against the applied potential, *E*, using AC voltammetry, until a stable curve was attained [9]. Incorporation of CSF114 and CSF114(Glc) into the tBLM was carried out by adding a small amount of its 1 mg/mL solution in DMSO to the working solution (buffered at pH 7 or unbuffered depending on the measurement under way), at an applied potential of −0.45 V. All measurements were carried out in aqueous 0.1 M KCl, and hence at a constant ionic strength of 0.1 M. The addition of 1 × 10^−3^ M HCl in the pH 3 solution and of about 1 × 10^−4^ M HCl and K_2_HPO_4_ in the pH 7 buffer solution do not alter the ionic strength to a significant extent.

Impedance spectroscopy and voltammetric measurements were carried out with an Autolab instrument PGSTAT12 (Echo Chemie, Utrecht, The Netherlands) supplied with FRA2 module for impedance measurements, SCAN-GEN scan generator and GPES 4.9007 software. Electrochemical impedance spectra at DPTL/DOPC and DPTL/DOPS tBLMs were recorded over the potential range from −0.25 to −0.95 V by varying the frequency from 10^5^ to 0.1 Hz.

## 4. Conclusions 

The present measurements indicate that the structure-based designed synthetic antigenic probe type I’ *β*-turn *N-*glucosylated peptide CSF114(Glc), devised to identify autoantibodies biomarkers of a multiple sclerosis patients’ subpopulation, may interact with the polar heads of the outer leaflet of biomembranes at moderate transmembrane potentials. In particular, it interacts more effectively with the polar heads of phosphatidylcholine than with those of phosphatidylserine at pH values less than 7. It is also adsorbed on top of microdomains present in membranes rich in sphingomyelin, one important component of the myelin sheath, whose proteins are believed to undergo an aberrant *N-*glucosylation responsible for an antibody-mediated form of multiple sclerosis. However, CSF114(Glc) does not form channel-like holes in lipid bilayers. These data allow us to speculate that early bacterial and/or viral infections can trigger aberrant *N-*glucosylation as a consequence of an inflammatory event exposing myelin protein antigens.
